# Synergistic inhibition effect of TNIK inhibitor KY-05009 and receptor tyrosine kinase inhibitor dovitinib on IL-6-induced proliferation and Wnt signaling pathway in human multiple myeloma cells

**DOI:** 10.18632/oncotarget.17056

**Published:** 2017-04-12

**Authors:** Yura Lee, Jung-Il Jung, Kyeong-Yong Park, Soon Ae Kim, Jiyeon Kim

**Affiliations:** ^1^ Department of Biomedical Laboratory Science, School of Medicine, Eulji University, Daejeon 34824, Korea; ^2^ R&D Center, Peptron, Inc., Daejeon 34054, Korea; ^3^ Department of Pharmacology, School of Medicine, Eulji University, Daejeon 34824, Korea; ^4^ Present address: Severance Biomedical Science Institute, Brain Korea 21 PLUS Project for Medical Science, College of Medicine, Yonsei University, Seoul 03722, Korea

**Keywords:** multiple myeloma, TNIK, KY-05009, IL-6, Wnt signaling

## Abstract

Multiple myeloma is a fetal form of plasma cell malignancy characterized by abnormal clonal proliferation of plasma cells. Especially, the canonical Wnt signaling pathway mediated by β-catenin is activated in multiple myeloma cells, stimulating their proliferation. Here, we investigated the relationship between interleukin-6-induced proliferation of multiple myeloma cells and Traf2- and Nck-interacting kinase (TNIK) expression in Wnt signaling. Interleukin-6 increased the proliferation of multiple myeloma cells and *TNIK* mRNA and protein expression. In addition, we examined the effect on TNIK of TNIK inhibitor KY-05009 and receptor tyrosine kinase inhibitor dovitinib and whether inhibition of TNIK suppresses the interleukin-6-induced proliferation of multiple myeloma cells. KY-05009 and dovitinib synergistically inhibited interleukin-6-stimulated proliferation and induced apoptosis through the inhibition of Wnt signaling in MM cells. Our results provide crucial information that TNIK is involved in the interleukin-6-dependent proliferation of multiple myeloma cells and inhibition of Wnt signaling involving TNIK could be a therapeutic strategy for the treatment of interleukin-6-dependent multiple myeloma.

## INTRODUCTION

Multiple myeloma (MM) is a plasma cell malignancy characterized by excess clonal proliferation of abnormal plasma cells in the bone marrow, elevated secretion of monoclonal proteins in the serum or urine, and multiple organ damage, including renal failure, hypercalcemia, anemia, and lytic bone resorption [1]. MM is estimated to account for approximately 10% of all hematological malignancies but remains an incurable hematological disorder, despite advances in the introduction of new drugs and therapies [2]. New drugs, such as thalidomide, lenalidomide, and bortezomib, have been used for the treatment of relapsed and refractory MM, but the median survival has remained 5-6 years in the elderly [3]. To increase the survival rate in MM patients, new therapeutic strategies using novel drugs are needed.

The serine/threonine kinase Traf2- and Nck-interacting kinase (TNIK), a member of the germinal center kinase (GCK) family, was first identified as a regulatory kinase in cell spreading or migration through cytoskeleton organization [4, 5]. In the last 10 years, TNIK has been reported as a novel therapeutic target in several types of cancers. In many studies, the expression of TNIK has been shown to be involved in the survival of human cancer cells, including colorectal, gastric, liver, and hematological cancer [6–11]. Wnt signaling is mediated by TNIK through interactions with β-catenin and following phosphorylation of T-cell factor (TCF) 4 at serine 154 in the nucleus. Phosphorylation of TCF4 induces the activation of Wnt target genes, such as *CCND1*, *AXIN2*, *ZCCHC12*, and *TCF7* [6]. In addition, the inhibition of TNIK expression by small-interfering RNA (siRNA) suppresses the transcriptional activity of TCF/lymphoid enhancer-binding factor (LEF) and induces apoptosis [7, 11]. A few reports have demonstrated the expression of TNIK and cancer cell proliferation in several types of cancer, but the relevance of TNIK to hematological malignancies, especially MM, has not been sufficiently described [6–11].

In our previous studies, we investigated the apoptosis-inducing effect of tyrosine kinase inhibitor dovitinib and its inhibition of TNIK kinase activity and endogenous Wnt signaling in human MM cells [11]. TNIK is highly expressed in MM cells compared to normal peripheral blood mononuclear cells (PBMCs), and inhibition of TNIK expression by siRNA induces cell death. KY-05009 and dovitinib have a high affinity for the ATP binding site in TNIK and inhibit the protein expression of TNIK and transcriptional activity of Wnt target genes [11, 12]. Through these our recent reports, we confirmed that TNIK can be a potential target for inducing apoptosis activity of KY-05009 and dovitinib in cancer cells.

In the present study, we investigated the level of TNIK expression in human MM cells from patients and the apoptosis-inducing effect of KY-05009 and dovitinib in the IL-6-dependent MM RPMI8226 cell line. IL-6 enhanced cell proliferation, *TNIK* mRNA and protein expression, and the transcriptional activity of Wnt target genes. KY-05009 exerted synergistic anti-proliferative effects with dovitinib and triggered caspase-dependent apoptosis in RPMI8226 cells. We hypothesize that a possible mode of action of KY-05009 and dovitinib is a high affinity for TNIK and subsequent inhibition of kinase activity [11, 12]. This inhibitory effect against TNIK may suppress the proliferation of RPMI8226 cells. Thus, our results suggest that TNIK could be an anti-cancer target for the investigation of treating MM by inhibiting Wnt signaling-mediated MM cell proliferation.

## RESULTS

### IL-6 stimulates the proliferation of RPMI8226 cells

**Figure 1:**
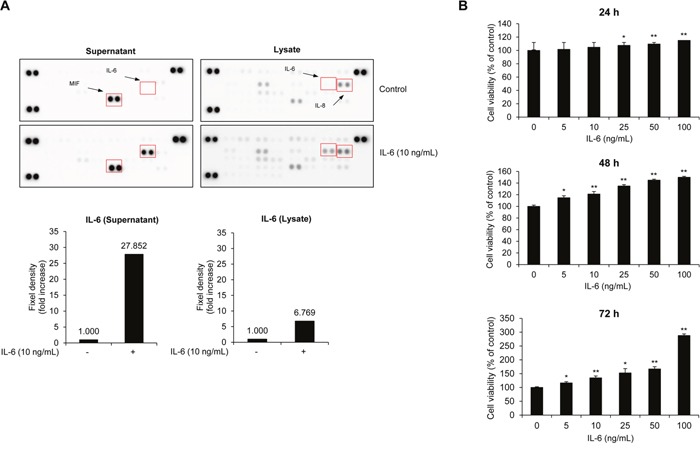
IL-6 activates MM cell proliferation **(A)** RPMI8226 cells were treated with recombinant human IL-6 for 72 h. After incubation, cytokine expression in cell supernatants and lysates was analyzed by human cytokine array. The expression of IL-6 was normalized by the density of control spots. **(B)** Cell viability of RPMI8226 cells after treatment with IL-6 (0-100 ng/mL) in serum-free medium for 24-72 h. Data are presented as mean±SD. The experiments were performed in triplicate. *P < 0.01, ^*^P < 0.001 versus control.

IL-6 has been identified as a major growth factor for myeloma cell proliferation *in vivo* and *in vitro* [13–16]. In particular, paracrine regulation of IL-6 stimulates myeloma cell proliferation in patients [13]. To confirm the effect of IL-6 on the production of cytokines in MM cells, we analyzed the expression of cytokines and whether IL-6 treatment induces paracrine effects of other cytokines, such as IL-1, IL-2, IL-4, IL-5, and tumor necrosis factor (TNF)-α, on cultured supernatant or protein expression in cell lysates (Figure 1A). Serum-starved RPMI8226 cells were treated with recombinant human IL-6 in serum-free medium for 72 h and the culture supernatants and cell lysates isolated to analyze secreted factors and their influence on protein expression in MM cells. A human cytokine array showed that migration inhibitor factor (MIF), an inflammatory mediator, was constitutively produced regardless of IL-6 treatment. IL-6 was only expressed in the supernatant in response to IL-6 treatment (Figure 1A, left). We also observed that IL-8, an activator of osteoclast differentiation and bone resorption in MM, was expressed in both untreated controls and the IL-6-treated group, but IL-6 only increased in the IL-6-treated lysate group (Figure 1A, right). Next, we assessed the stimulatory effect of IL-6 on the proliferation of MM cells. RPMI8226 cells were incubated with recombinant human IL-6 for 24 to 72 h. As shown in Figure 1B, IL-6 stimulated the proliferation of MM cells in a dose- and time-dependent manner. These results support an increased level of IL-6 in cultured supernatants and cell lysates correlating with MM cell proliferation.

### IL-6 activates TNIK expression and the transcriptional activity of Wnt signaling

**Figure 2:**
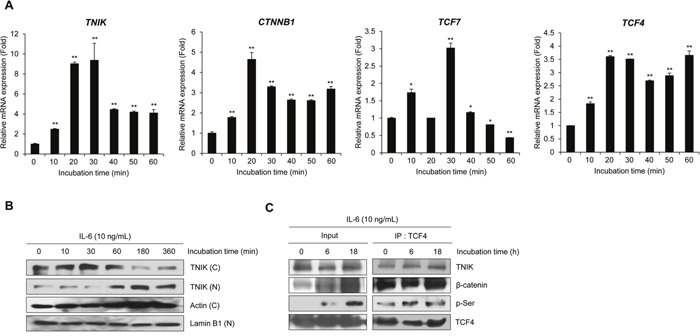
IL-6 activates TNIK expression and Wnt target gene transcription **(A)** qRT-PCR analysis of the indicated genes in serum-starved RPMI8226 cells after treatment with IL-6 (10 ng/mL) for 0-30 min. Data are presented as mean±SD. Experiments were performed in triplicate. *P < 0.01, ^*^P < 0.001 versus control. **(B)** Western blot of TNIK expression in the cytosolic **(C)** and nuclear (N) fractions of RPMI8226 cells treated with IL-6 (10 ng/mL) in serum-free medium for 0 – 360 min. Actin was used as a loading control. **(C)** Phosphorylation of TCF4 and expression of TCF4-bound proteins were measured by immunoprecipitation and western blot analysis of RPMI8226 cells treated with IL-6 (10 ng/mL) in serum-free medium for 0, 6, and 18 h.

Our previous studies demonstrated the association between canonical Wnt signaling and the survival of MM cells [17, 18]. In addition, targeting of the constitutively active β-catenin/TCF4 transcriptional complex has been identified as a potent therapeutic strategy in the treatment of MM [19]. Several reports have shown that TNIK activates the transcription of Wnt target genes through interactions with β-catenin and TCF4 and following the phosphorylation of TCF4 at serine 154 [6, 7, 11]. In this study, we confirmed the IL-6-induced transcriptional activity of Wnt target genes and TNIK protein expression using RPMI8226 cells. First, we determined *TNIK* mRNA expression in human MM patients (n=15) (Supplementary Figure 1). The relative endogenous *TNIK* mRNA expression was increased compared to primary PBMCs. To evaluate the relationship between IL-6 and the activation of TNIK expression and Wnt signaling, we detected the IL-6-induced transcriptional activity of TNIK and Wnt target genes. As shown in Figure 2A and Supplementary Figure 2, IL-6 treatment activated *TNIK* mRNA expression and Wnt signaling-related genes, including *CTNNB1*, *TCF4*, and *TCF7* until 60 minutes (Table 1).

**Table 1:** Primer sequences used in this study

Target gene	Forward (5’ – 3’)	Reverse (5’ – 3’)
*TCF7*	CTGCACATGCAGCTATACCC	GGCCACCTGTCTCTGAGATT
*CTNNB1*	CCATCTTCCAGGAGCGAGAT	CAGTGATGGCATGGACTGTG
*TCF4*	GAGCAGCAAGTCCGAGAAAG	ATGCTGAAACCTCTTGCGTC
*TNIK*	GCTATTGAGATCCGGTCAGT	CAGGCTGCAACATTGAAAGA
*GAPDH*	GAGTCAACGGATTTGGTCGT	GATCTCGCTCCTGGAAGATG

IL-6 treatment induced translocation of cytosolic TNIK into the nucleus in a time-dependent manner (Figure 2B). We also used co-immunoprecipitation to confirm the effect of IL-6 on the activation of Wnt signaling. As shown in Figure 2C, IL-6 treatment induced the phosphorylation of TCF4. These results indicate that IL-6-induced MM cell proliferation is related to the expression of TNIK and activation of the Wnt signaling pathway in MM cells.

### Inhibition of TNIK induces caspase-dependent apoptosis in MM cells

**Figure 3:**
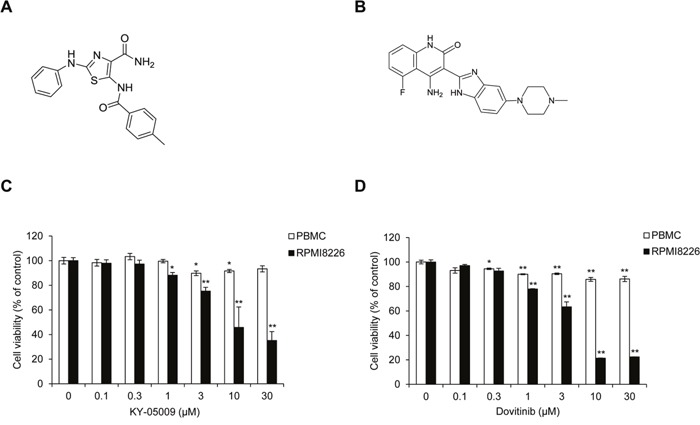
KY-05009 and dovitinib inhibit MM cell proliferation **(A)** Chemical structure of KY-05009. **(B)** Chemical structure of dovitinib. **(C and D)** Cell viability of peripheral blood mononuclear cells (PBMCs) and RPMI8226 cells treated with KY-05009 **(C)** or dovitinib **(D)** in RPMI1640 medium containing 5% FBS for 24 h. Data are presented as mean±SD. Experiments were performed in triplicate.*P < 0.01, ^*^P < 0.001 versus control.

In a previous study, we found that the inhibition of TNIK by dovitinib or siRNA suppresses the expression of endogenous Wnt signaling regulatory proteins and MM survival [11]. We also confirmed that a novel aminothiazole TNIK inhibitor, KY-05009 (5-(4-methylbenzamido)-2-(phenylamino) thiazole-4-carboxamide), has high affinity for the ATP binding site of TNIK and pharmacologically inhibits transforming growth factor (TGF)-β1-induced epithelial-to-mesenchymal transition (EMT) in human lung adenocarcinoma cells [12]. In MM cells, dovitinib exerts anti-cancer activity through inhibition of TNIK kinase activity and the endogenous Wnt signaling pathway [11]. To confirm the association between TNIK inhibition and IL-6-induced MM cell proliferation, we assessed the viability of RPMI8226 cells using KY-05009 (Figure 3A) and dovitinib (Figure 3B). As shown in Figure 3C and 3D, KY-05009 and dovitinib inhibited the proliferation of RPMI8226 cells, but there was also significant cytotoxicity in normal PBMCs.

**Figure 4:**
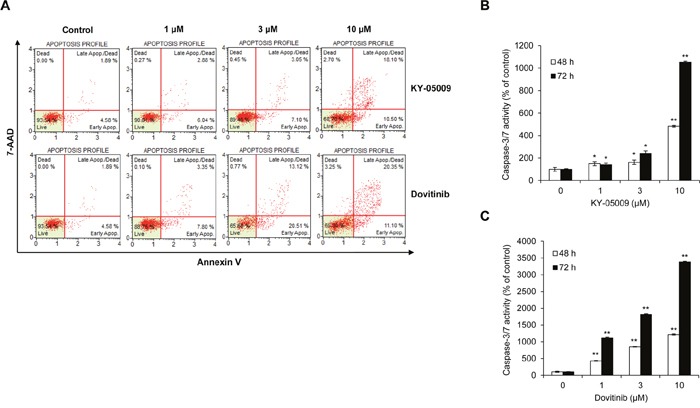
KY-05009 and dovitinib induce apoptosis of MM cells **(A)** Flow cytometry results for RPMI8226 cells treated with KY-05009 or dovitinib for 48 h. The apoptosis profile was represented by Annexin V and 7-AAD uptake (Annexin V versus 7-AAD). Each reported result is representative of triplicate experiments. **(B and C)** RPMI8226 cells were treated with KY-05009 **(B)** or dovitinib **(C)** for 48 or 72 h and the relative caspase-3/7 activity measured. Data are presented as mean±SD. Experiments were performed in triplicate. *P < 0.01, ^*^P < 0.001 versus control.

Next, to determine the cell death–inducing mechanism of KY-05009 and dovitinib, we used flow cytometry to evaluate apoptosis. KY-05009 and dovitinib induced the binding of fluorescent Annexin V and 7-amino-actinomycin D (7-AAD) uptake, key features of late apoptosis, in a dose-dependent manner (Figure 4A) [20–22]. KY-05009 and dovitinib induced apoptosis at concentrations over 1 μM (Figure 4B and 4C). This result suggests that inhibition of TNIK by kinase inhibitors could contribute to the induction of apoptosis of MM cells.

### KY-05009/dovitinib combination treatment synergistically induces apoptosis of MM cells

**Figure 5:**
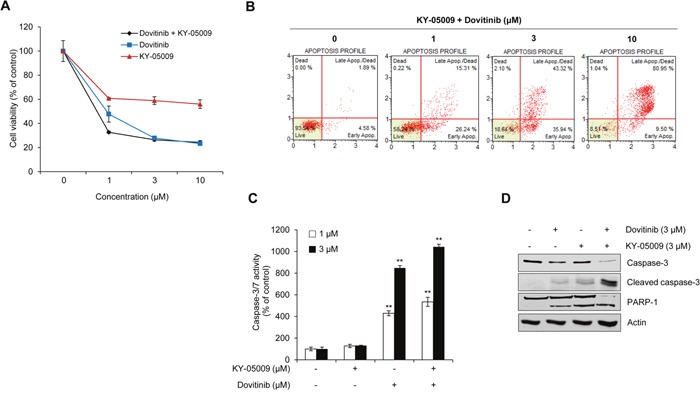
Synergistic effect of KY-05009 and dovitinib induces apoptosis of MM cells **(A)** RPMI8226 cells were treated with KY-05009 alone or combination with dovitinib (0-10 μM) for 48 h and cell viability measured. **(B)** The apoptotic population of KY-05009 and dovitinib co-treated cells was analyzed by flow cytometry. Each reported result is representative of triplicate experiments. **(C)** RPMI8226 cells were treated with KY-05009 and dovitinib (1 or 3 μM) for 48 h and the relative caspase 3/7 activity measured. Data are presented as mean±SD. Experiments were performed in triplicate. ^*^P < 0.001 versus control. **(D)** Western blot of cleaved caspase-3 expression in the cytosolic fraction and PARP-1 in the nuclear fraction of RPMI8226 cells treated with KY-05009 or dovitinib alone or in combination for 48 h. Actin was used as a loading control.

We assessed cell viability and apoptosis in the presence of TNIK inhibition to determine whether KY-05009 and dovitinib cause synergistic effects in RPMI8226 cells (Figure 5A). The combination index (CI) was also calculated (Table 2). Compared to the single treatment results, combined treatment had a synergistic effect. To evaluate whether this effect was due to apoptosis, we analyzed the population of early and late apoptotic cells using flow cytometry. As shown in Figure 5B, combined treatment had a synergistic apoptosis-inducing effect compared to the single compound treatment in Figure 4A. Furthermore, combined treatment increased the caspase-3/7 activity (Figure 5C) and cleavage of downstream apoptosis-related substrate poly (ADP-ribose) polymerase 1 (PARP-1) in the nucleus (Figure 5D). These results suggest that inhibition of TNIK using specific TNIK inhibitor KY-05009 and tyrosine kinase inhibitor dovitinib could have a synergistic effect on the induction of apoptosis of MM cells.

**Table 2:** Combination index (CI) values for the two-drug combination against RPMI8226 cell viability

KY-05009 (μM)	Dovitinib (μM)	CI value
1	1	0.2210
1	3	0.3428
1	10	0.7951
3	1	0.1971
3	3	0.3661
3	10	0.7778
10	1	0.1939
10	3	0.2906
10	10	0.7474

### KY-05009/dovitinib combination treatment synergistically inhibits IL-6-induced proliferation and activation of Wnt signaling

**Figure 6:**
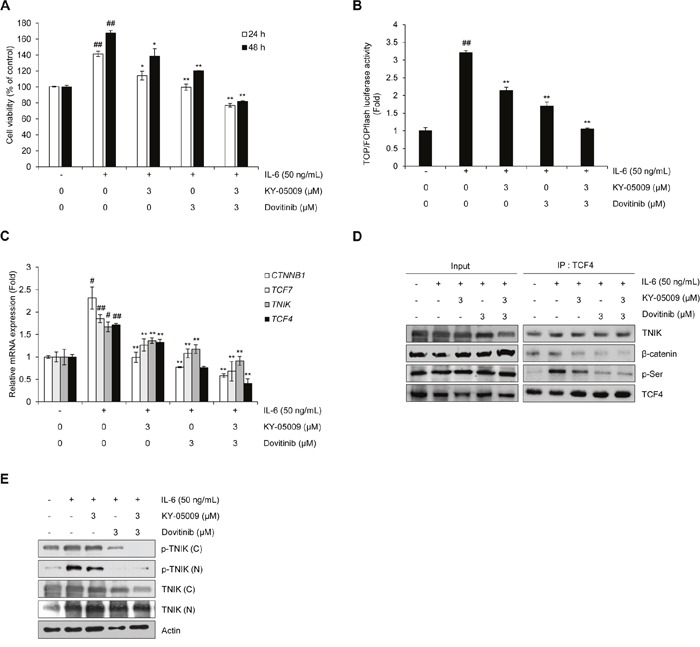
Inhibition of TNIK suppresses IL-6-induced proliferation and the Wnt signaling pathway in MM cells **(A)** Cell viability of RPMI8226 cells treated with IL-6 (50 ng/mL) and KY-05009 (3 μM) or dovitinib (3 μM) alone or in combination for 24 or 48 h. Data are presented as mean±SD. Experiments were performed in triplicate. **(B)** Relative TCF/LEF luciferase activity measured by FOPflash-normalized TOPflash luciferase activityin RPMI8226 cells treated with IL-6 (50 ng/mL) and KY-05009 (3 μM) or dovitinib (3 μM) alone or in combination for 9 h. **(C)** RPMI8226 cells treated with IL-6 (50 ng/mL) and KY-05009 (3 μM) or dovitinib (3 μM) alone or in combination for 1 h. The mRNA expression of indicated genes was detected by qRT-PCR analysis. **(D and E)** The expression of TCF4-interacting proteins and phosphorylation of TNIK detected by immunoprecipitation and Western blot of RPMI8226 cells treated with IL-6 (50 ng/mL) and KY-05009 (3 μM) or dovitinib (3 μM) alone or in combination for 9 h. *P < 0.01, ^*^P < 0.001 versus control; ^#^ *P* < 0.01, ^##^*P* <0.001 versus cells treated with IL-6 alone.

To confirm the synergistic inhibitory effect of KY-05009 and dovitinib on IL-6-induced activation of Wnt signaling in MM cells, we determined cell viability and performed a TOP/FOPflash luciferase assay. RPMI8226 cells were incubated with IL-6 alone or KY-05009 and/or dovitinib for 24 or 48 h. As shown in Figure 6A, IL-6 alone increased the cell proliferation rate, but KY-05009, dovitinib, and the combination of both decreased cell viability in a dose-dependent manner. The inhibitory effect of this combination treatment on cell proliferation was confirmed using other multiple myeloma cell lines such as IM-9 (IL-6-independent) and MM.1R (IL-6-dependent) cells (Supplementary Figure 3 and Supplementary Figure 4, Supplementary Table 1 and Supplementary Table 2). We also assessed the transcriptional activity of TCF4 using TOP/FOPflash reporter luciferase assay to determine whether inhibition of TNIK using KY-05009 and dovitinib suppresses the IL-6-induced activation of Wnt signaling. As shown in Figure 6B, KY-05009 and dovitinib inhibited the IL-6-induced TCF4-mediated transcription at 3 μM, and combination treatment significantly inhibited TCF4-mediated transcriptional activity. We also examined the TNIK protein expression and transcriptional activity of Wnt target genes described in a previous report [6, 7, 11]. IL-6 increased the mRNA expression of Wnt signaling-related genes, including *TNIK*, *CTNNB1*, *TCF7*, and *TCF4*, but KY-05009 and dovitinib suppressed the transcriptional activity of these genes (Figure 6C).

We confirmed the synergistic effect of KY-05009 and dovitinib through immunoprecipitation. As shown in Figure 6D, IL-6 treatment time of 9 h increased the phosphorylation of TCF4. The IL-6-induced interaction between TCF4 and β-catenin and the phosphorylation of TCF4 were inhibited by KY-05009 or dovitinib or combined treatment. Although 9 h of treatment did not significantly inhibit TNIK expression, TNIK expression and phosphorylation in cytosol and nuclear fractions were inhibited by KY-05009 and dovitinib after 18 h treatment (Figure 6E). These results indicate that the inhibitory effect of KY-05009 and dovitinib could contribute to the IL-6-induced proliferation of MM cells through the suppression of TNIK-mediated Wnt signaling.

## DISCUSSION

In MM, a canonical Wnt/β-catenin signaling pathway is endogenously activated or stimulated with Wnt ligands, increasing MM cell survival [23]. TNIK was introduced as an important transcriptional mediator in the canonical Wnt signaling pathway [6, 7]. In addition, overexpression of nuclear TNIK is related to the prognosis of cancer [9]. These previous studies indicate that TNIK-mediated activation of Wnt signaling could be a therapeutic target in the development of anti-cancer agents.

Silencing of endogenous TNIK and inhibition of TNIK kinase activity suppressed the proliferation of MM cells and induced caspase-dependent apoptosis [11]. Dovitinib also inhibited the expression of TNIK and the interaction with TCF4 in the nucleus. Phosphorylation of TCF4 was suppressed by dovitinib treatment, which then inhibited the transcriptional activity of Wnt target genes. These results provide crucial information that the inhibition of endogenous TNIK induces the suppression of Wnt signaling regulating MM cell survival.

IL-6 has been identified as a major growth factor for the differentiation of B cells to antibody-producing plasma cells and in myeloma cell growth [13–16, 24]. To investigate the stimulation effect of IL-6 on cell proliferation and TNIK expression in Wnt signaling, we used the RPMI8226 MM cell line, which responds to exogenous IL-6 treatment but does not express IL-6 or IL-6 receptor transcripts [24]. Based on our recent report, we hypothesized that targeting TNIK and the Wnt signaling pathway may be a novel therapeutic approach in the development of new drugs for MM treatment. Exogenous IL-6 did not significantly activate the production of other cytokines in MM cells, but increased concentrations of IL-6 dose- and time-dependently stimulated the proliferation of MM cells. Similarly, IL-6 treatment increased the mRNA expression of Wnt target genes and TNIK protein expression. These results suggest that IL-6-induced activation of TNIK-mediated Wnt signaling influences MM cell proliferation.

KY-05009 is a novel aminothiazole developed as a TNIK inhibitor that exhibits anti-cancer activity in human colorectal cancer cells [25]. KY-05009 had high affinity for the ATP binding site of TNIK that plays a crucial role in the inhibition of kinase activity (*K*_i_ = 100 nM) [12]. In addition, KY-05009 inhibited the interaction between TCF4 and TNIK/β-catenin that suppressed the phosphorylation of TCF4. This report provides pivotal information that the inhibition of TNIK and Wnt signaling by KY-05009 can be a new therapeutic strategy for the treatment of Wnt signaling-activated cancer cells. Our study also suggested that inhibition of Wnt signaling by TNIK inhibitors can suppress the IL-6-induced proliferation of MM cells. The anti-cancer activity of KY-05009 was elevated by co-treatment with dovitinib, a selective receptor tyrosine kinase inhibitor. Although dovitinib exerts inhibitory effects on various kinases, it also exhibits high binding affinity for the ATP binding site of TNIK with an inhibiting effect on the kinase activity [11, 26–28]. We confirmed that dovitinib has an inhibitory effect on the expression of Wnt signaling modulators, such as β-catenin and TCF4, as well as TNIK, in MM cells [11]. Previous studies support our results that inhibition of TNIK by treatment with both KY-05009 and dovitinib exerts synergistic effect on the proliferation of MM cells.

Recent studies have described the potential of TNIK as an anti-cancer target to inhibit Wnt signaling in several types of human cancers [4, 6–11] and TNIK inhibitors have been developed to treat Wnt-active cancers, but more experimental evidence is needed to elucidate the mechanism underlying the inhibition of proliferation in cancers. Although the biological function of TNIK in hematological cancers, such as MM and leukemia, has not been clearly investigated yet, our results suggest a new approach for treating MM stimulated by exogenous IL-6. Based on our results, we hypothesize that exogenous IL-6, in a paracrine or autocrine manner, elevates the expression of TNIK and the subsequent activation of Wnt signaling, which enhances MM cell survival. In particular, our results show that the combinatorial use of existing kinase inhibitors with an inhibitory effect on TNIK kinase activity and TNIK inhibitors may be an effective therapeutic strategy for the treatment of MM. As a rare hematological disease, the methods for treating MM are still limited, but increasing data on TNIK and its biological function in human cancers will be crucial for the development of TNIK-targeting drugs.

## MATERIALS AND METHODS

### Cell culture and PBMC preparation

The human MM RPMI8226 cell line was obtained from the Korean Cell Line Bank (#10155). The use of human primary PBMCs and MM patient's blood samples for this study was approved by the International Review Board of Eulji University (EU 16-10). All cells were maintained in RPMI-1640 medium (Corning Inc., NY, USA) containing 5% fetal bovine serum (FBS) (Corning Inc., NY, USA) and 1% antibiotics (100 U/mL penicillin and 100 μg/mL streptomycin, HyClone™, GE Healthcare, Salt Lake City, UT, USA) in a humidified atmosphere of 5% CO_2_ at 37°C.

### Cell viability assay

PBMCs or RPMI8226 cells (1.0×10^4^ cells/well) were seeded in 96-well plates and incubated for 24 h. After incubation, the cells were treated with dovitinib (Selleck Chemicals, USA) and/or KY-05009 [12] in complete medium containing 5% FBS for 24-72 h. Cell viability was measured using the Cell Counting Kit-8 (Dojindo Molecular Technologies, Kumamoto, Japan) according to the manufacturer's instructions. The absorbance was measured using the Multiscan™ FC microplate photometer (Thermo Fisher Scientific, Boston, MA, USA). Experiments were performed in triplicate.

### Human cytokine array

The cytokine levels after stimulation with human IL-6 were measured using the Proteome Profiler™ Cytokine Array Kit (R&D Systems, MN, USA). RPMI8226 cells were seeded in 60-mm^2^ dishes in culture medium containing 5% FBS for 24 h. The cells were replenished with serum-free medium for 24 h before treatment with recombinant human IL-6 (10 ng/mL) (R&D Systems, MN, USA) for 18 h. After incubation, all experimental procedures were completed according to the manufacturer's instructions.

### Flow cytometry

RPMI8226 cells (1×10^5^ cells/mL) were seeded in 6-well plates and treated with KY-05009 and/or dovitinib (0–10 μM) in complete medium containing 5% FBS for 48 h. Harvested cells were washed with phosphate-buffered saline (PBS) and treated using the MUSE™ Annexin V Dead Cell Assay Kit (Merck Millipore, Darmstadt, Germany). The population of apoptotic and dead cells was determined using the MUSE™ Cell Analyzer (Merck Millipore, Darmstadt, Germany) and acquired data analyzed using the MUSE™ Annexin V and Dead Cell software module (Merck Millipore, Darmstadt, Germany) according to the manufacturer's instructions.

### Western blot analysis

Cytoplasmic and nuclear fractions were prepared from RPMI8226 cell lysates using NE-PER nuclear and cytoplasmic extraction reagent (Thermo Fisher Scientific, Boston, MA, USA). After protein quantification, cytoplasmic or nuclear proteins were loaded on polyacrylamide gels for electrophoresis and the separated proteins transferred to nitrocellulose membranes. Protein expression was detected using specific primary antibodies against p-TNIK, TNIK, TCF4, Lamin B1, β-catenin, and actin (Santa Cruz Biotechnology, Inc., Austin, TX, USA); PARP-1, caspase-3, and cleaved caspase-3 (Cell Signaling Technology, Inc., Danvers, MA, USA); and phosphoserine (p-Ser; Abcam, Cambridge, UK). After incubation with horseradish peroxidase (HRP)-conjugated secondary antibodies, the signals were visualized using Luminata™ Forte Western HRP Substrate (Merck Millipore, Darmstadt, Germany). The band intensities were measured to determine the relative protein expression using X-ray films and development solution (Fujifilm, Tokyo, Japan). Bands were quantified using ImageJ software. Actin was used as a loading control.

### Immunoprecipitation

Total lysates of RPMI8226 cells were pre-cleared with normal control immunoglobulin G and Protein A agarose beads (Thermo Fisher Scientific, Boston, MA, USA) after protein quantification. Endogenous protein complexes were immunoprecipitated overnight at 4°C with an anti-TCF4 antibody (Santa Cruz Biotechnology, Inc., Austin, TX, USA) plus Protein A agarose beads. TCF4-bound proteins were subjected to 4%–20% polyacrylamide gel electrophoresis and Western blot performed. TCF4-bound proteins were detected using specific primary antibodies against TNIK, p-Ser, and β-catenin. After incubation with HRP-conjugated secondary antibodies, the signals were visualized using Luminata™ Forte Western HRP Substrate (Merck Millipore, Darmstadt, Germany). The band intensities were measured to determine the relative protein expression using X-ray films and development solution (Fujifilm, Tokyo, Japan).

### TOP/FOPflash reporter luciferase assay

RPMI8226 cells were trasfected with TOPflash TCF reporter plasmid (wild-type TCF binding site) (Merck Millipore, Darmstadt, Germany), FOPflash plasmid (mutant TCF binding site) (Merck Millipore, Darmstadt, Germany), and Lipofectamine^2000^ (Thermo Fisher Scientific, Boston, MA, USA) in an antibiotic-free medium. FOPflash-normalized TOPflash luciferase activity was represented as the relative TCF/LEF luciferase activity. After TOP/FOPflash reporter transfection, serum-deprived cells were treated with IL-6 (50 ng/mL) and KY-05009, dovitinib, or both for 48 h. Cells were washed with PBS, and then lysed using passive lysis buffer (Promega, Madison, WI, USA). The luciferase activity was evaluated using the luciferase reporter assay (Promega, Madison, WI, USA).

### Measurement of caspase-3/7 activity

RPMI8226 cells (2×10^5^ cells/mL) were treated with dovitinib and/or KY-05009 (1 or 3 μM) in RPMI-1640 medium containing 5% FBS for 48 h. After cell harvesting, caspase-3/7 activity was measured using Caspase-Glo^®^ 3/7 Assay Systems (Promega, Madison, WI, USA). Detailed experiments were performed as described in the manufacturer's instructions.

### Quantitative real-time RT-PCR (qRT-PCR)

Total RNA was isolated from the frozen buffy coat specimens of human MM patients using the NucleoSpin^®^ RNA Blood extraction kit (Macherey-Nagel, GmbH&Co. KG, Germany). RPMI8226 cells were treated with IL-6 (10 ng/mL) in RPMI-1640 medium containing 0.1% FBS for 0 - 60 min. After incubation, the total RNA was isolated from RPMI8226 cells using the AccuPrep® RNA Extraction Kit (Bioneer Corp., Daejeon, Korea) and the cDNA synthesized from 1 μg of total RNA using oligo (dT) primers (Bioneer Corp., Daejeon, Korea) and the RocketScript™ Reverse Transcriptase Kit (Bioneer Corp., Daejeon, Korea). Quantitative real-time RT-PCR was performed using ExcelTaq 2X Q-PCR Master Mix (SMOBiO, Hsinchu, Taiwan) and the CFX96™ Real-Time PCR System (Bio-Rad, Sacramento, CA, USA). The cycling conditions were as follows: 95°C for 3 min followed by 40 cycles at 95°C for 15 s, 60°C for 30 s, and 72°C for 30 s. Table 1 lists the target-specific primer sequences. All reactions were performed in triplicate with GAPDH as an internal standard. The data were analyzed using the 2^−ΔΔC^_T_ method [29].

### Analysis of combined drug effects

The effect of the combined drugs was analyzed using the Calcusyn software program (Biosoft, Cambridge, UK) and CI methods derived from the median effect principle of Chou and Talalay [30] to determine whether the combined treatment of two compounds was additive or synergistic. The CI was calculated by the formula [31]; a CI of 1 indicated an additive effect between the two compounds, whereas a CI > 1 or CI < 1 indicated antagonism or synergism, respectively.

### Statistical analysis

All data are reported as mean±SD. Significant differences were determined by the Student's *t*-test.

## SUPPLEMENTARY MATERIALS FIGURES AND TABLES


