# Supplementary material for: Synergistic inhibition effect of TNIK inhibitor KY-05009 and receptor tyrosine kinase inhibitor dovitinib on IL-6-induced proliferation and Wnt signaling pathway in human multiple myeloma cells
Source: Oncotarget. 2017 Apr 12;8(25):41091–101. doi: 10.18632/oncotarget.17056 (PMC5522218; doi:10.18632/oncotarget.17056)
Supplement: Supplementary file 1 [file oncotarget-08-41091-s001.pdf]

Synergistic inhibition effect of TNIK inhibitor KY-05009 and receptor tyrosine kinase inhibitor dovitinib on IL-6-induced proliferation and Wnt signaling pathway in human multiple myeloma cells

SUPPLEMENTARY FIGURES

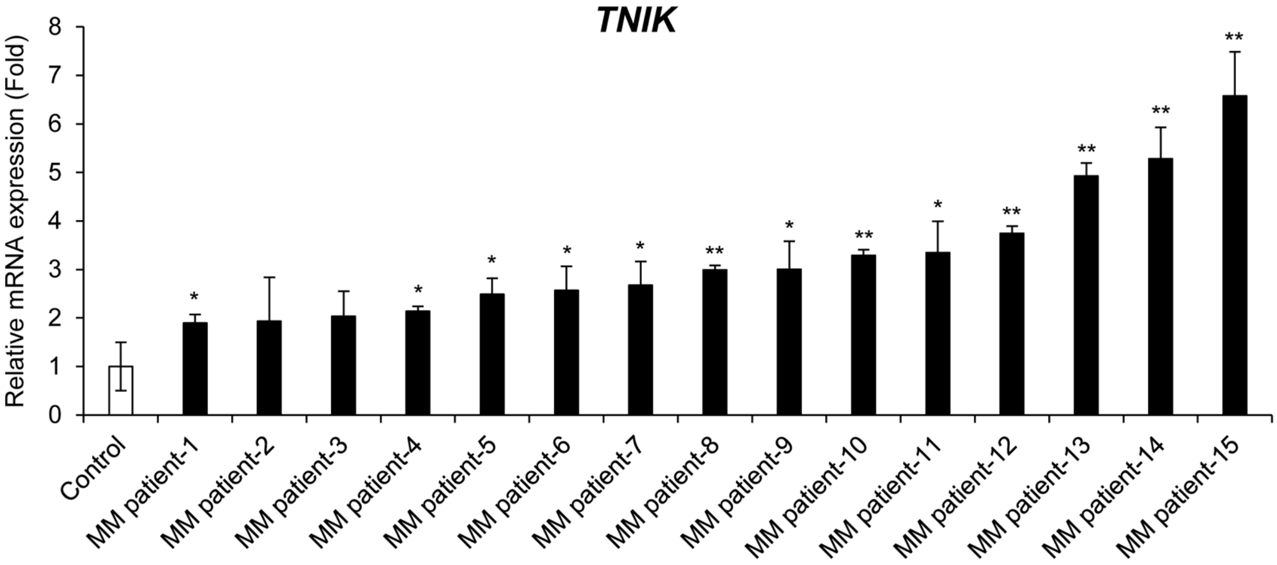

Supplementary Figure 1: *TNIK* mRNA expression in cells from MM patients. qRT-PCR analysis of the indicated genes using total RNA isolated from normal PBMCs (n=5) and MM patient's blood samples. The control group represents the average of qRT-PCR results obtained by PBMCs from 5 healthy individuals. Data are presented as mean±SD. Experiments were performed in triplicate. $^{*}P < 0.05$, $^{**}P < 0.01$ versus control.

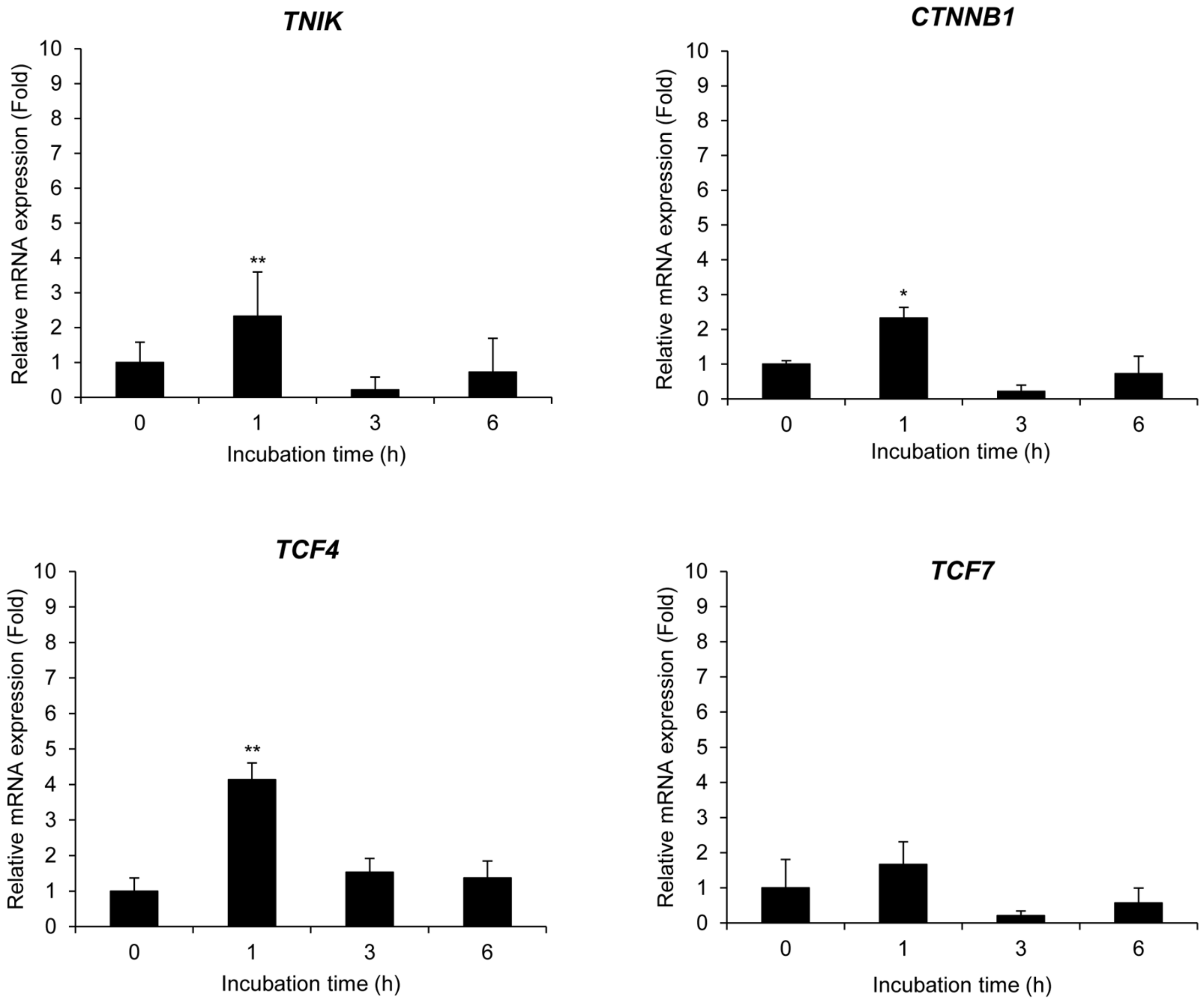

Supplementary Figure 2: The mRNA expression of Wnt target genes. qRT-PCR analysis of the indicated genes in serum-starved RPMI8226 cells after treatment with IL-6 (10 ng/mL) for 0-6 h. Data are presented as mean±SD. Experiments were performed in triplicate. $^{*}P < 0.01$, $^{**}P < 0.001$ versus control.

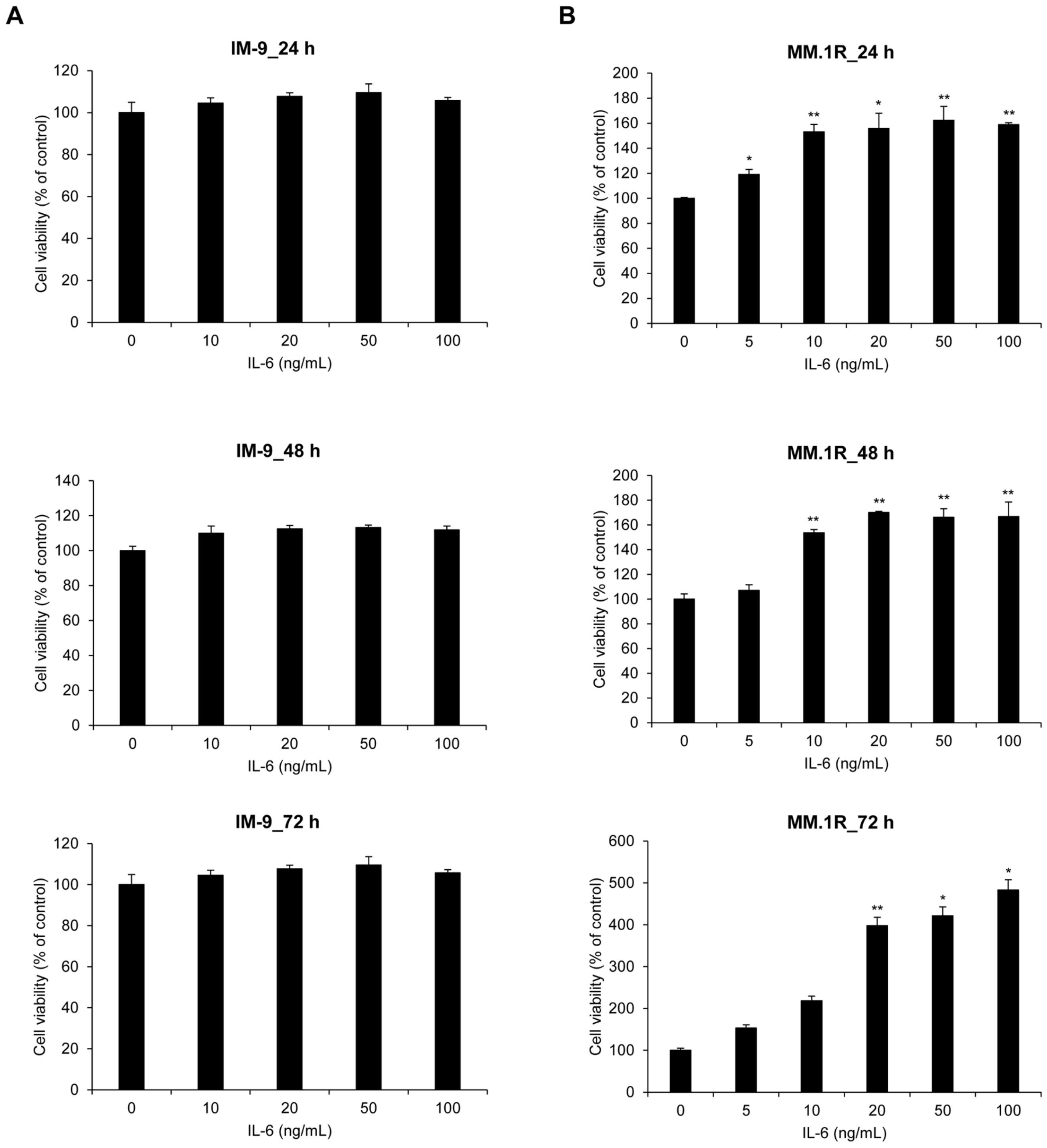

Supplementary Figure 3: Effect of IL-6 treatment on cell growth in MM IM-9 and MM.1R cells. **(A and B)** Cell viability of IM-9 **(A)** and MM.1R **(B)** cells after treatment with IL-6 (0-100 ng/mL) in serum-free medium for 24-72 h. Data are presented as mean±SD. The experiments were performed in triplicate. $^{*}P < 0.01$, $^{**}P < 0.001$ versus control.

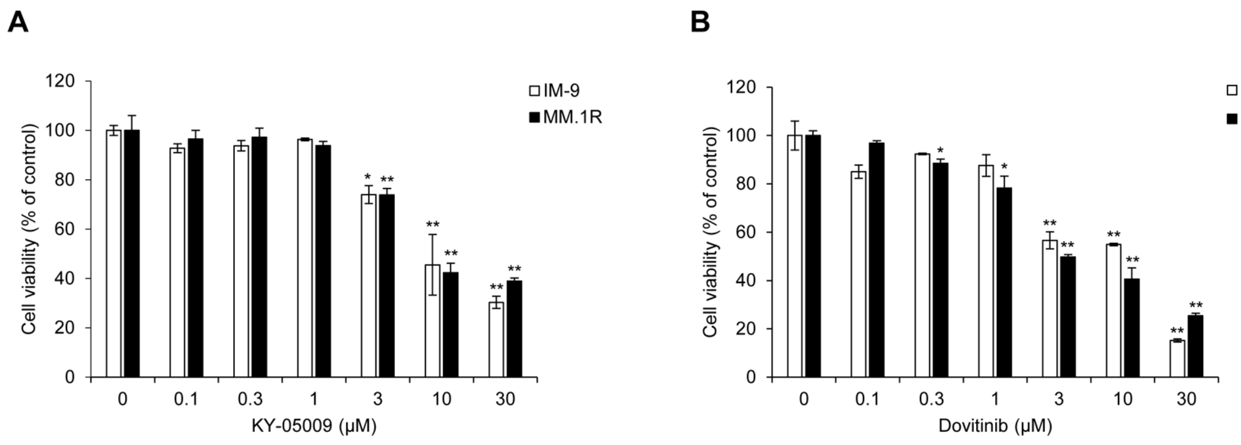

Supplementary Figure 4: KY-05009 and dovitinib inhibit MM IM-9 and MM.1R cell proliferation. **(A and B)** IM-9 and MM.1R cells were treated with cells treated with KY-05009 **(A)** or dovitinib **(B)** in RPMI1640 medium containing 5% FBS for 24 h. Data are presented as mean±SD. Experiments were performed in triplicate.$^{*}P < 0.01$, $^{**}P < 0.001$ versus control.

Supplementary Table 1: Combination index (CI) values for the two-drug combination against IM-9 cell viability

KY-05009 (μM)	Dovitinib (μM)	CI value
0.3	0.3	0.7438
1.0	1.0	0.9450
3.0	3.0	0.8818
10.0	10.0	0.3122
30.0	30.0	0.1833

Supplementary Table 2: Combination index (CI) values for the two-drug combination against MM.1R cell viability

KY-05009 (μM)	Dovitinib (μM)	CI value
0.3	0.3	0.5903
1.0	1.0	0.4356
3.0	3.0	0.7225
10.0	10.0	0.2754
30.0	30.0	0.4397
